# Functional Morphology and Defensive Behavior in a Social Aphid

**DOI:** 10.3390/insects10060163

**Published:** 2019-06-09

**Authors:** Junaid Ali Siddiqui, Xuting Zou, Qian Liu, Hui Zhang, Xiaolan Lin, Xiaolei Huang

**Affiliations:** State Key Laboratory of Ecological Pest Control for Fujian and Taiwan Crops, College of Plant Protection, Fujian Agriculture and Forestry University, Fuzhou 350002, China; junaidali206@gmail.com (J.A.S.); 18652038064@163.com (X.Z.); liuqian9502@163.com (Q.L.); zhanghui1903@163.com (H.Z.); linxl@fafu.edu.cn (X.L.)

**Keywords:** morphometry, eusocial aphid, deimatic display, phenotypic plasticity, Hemiptera

## Abstract

Social aphids produce different morphs, which are genetically identical but morphologically different. Each morph performs a different duty in its community. Social aphids usually produce morphologically distinct soldiers to protect their colonies. The social aphid *Pseudoregma bambucicola* produces sterile first instar soldiers with specialized body parts and unique defensive behaviors, such as hind leg waving. By using this species as a research model, this study tested the assumption that the functional morphological basis of defensive behaviors of soldiers is related to specialized body parts. Field observations and a comprehensive morphometric analysis were carried out for natural populations. The results showed significant differences in functional morphology between soldiers, first instar nymphs, and adults. Elongated hind legs in soldiers are an important functional morphological basis for the deimatic behavior of hind leg waving, while sclerotized front legs and head horns are related to the function of directly attacking natural enemies. The size variation of different body parts among different morphs also indicates a cost–benefit trade-off in the evolution of the social aphid species.

## 1. Introduction

Understanding the evolutionary process of morphological diversity and the associated costs and benefits are among the main objectives of evolutionary biology. Defensive approaches are critical components of animal survival, upon which life and death hinge. Mainly smaller animals employ various defensive tactics to elude predation [1]. Animals protect themselves by using primary and secondary defensive techniques. Various kinds of defensive strategies performed at different stages of life depend on the kind of threat. Primary defenses include different shapes, such as cryptic, warning coloration, and modified body parts. Secondary defenses can be in the form of feigning death, protean escape, chemical attack, crop fluid squirting, and deimatic behavior [2,3]. Deimatic behavior is a form of secondary defense behavior, in which animals scare their enemies by displaying a terrifying attitude as well as furious, startling, and various threatening patterns of behavior. Some insects, such as the praying mantis, larger eyed hawkmoths, *Smerinthus ocellatus*, and smaller peacock butterflies, display deimatic behavior [4]. To date, very few insects in Hemiptera have been reported to have such deimatic behavior [5].

*Pseudoregma bambucicola* (Takahashi) is a bamboo-feeding subtropical aphid belonging to the subfamily Hormaphidinae, which causes heavy infestation of the stem and shoots of bamboos [6]. This species produces first instar soldiers to protect their colonies [7]. The specialized soldier individuals are sterile and never molt to the second instar, while other normal first instar nymphs can mature into adults with regular reproduction [7,8]. The production of different types of larval morphs is called larval polymorphism [7,8,9,10]. The soldiers are significantly distinct from normal individuals due to their anatomical specialization—namely, a scorpion-like appearance, having well-developed frontal head horns with sharp tips, enlarged and robust forelegs, and sclerotized tergites. They protect the colony by attacking the eggs of natural enemies, such as syrphid fly larvae and small ladybird beetles [11]. As a primary defense behavior, soldiers of *P. bambucicola* swiftly grasp and attack the larvae of predatory moths and the adults of parasitoid wasps [12]. Sakata and Ito [13] also observed that when a colony is disturbed by breath tactile stimuli, the soldiers can wave their hind legs in retort to mechanical stimuli. They shake their legs in different ways that may depend on the kind of threat or the intensity.

Soldier aphids are not only restricted to Hormaphidinae, but are also produced in Eriosomatinae [12,14]. Most species produce soldiers on their primary hosts during the gall formation stage, whereas few species produce soldiers on their secondary hosts [15]. Usually, gall-forming aphids do not have soldiers with specialized body parts, but can be distinguished by their darker color and altruistic tasks (e.g., colony defense and gall repair). A social aphid such as *Tuberaphis styraci* (Hormaphidinae) produces first instar nymphs, which molt into second instar normal (yellow) and soldier (dark green) aphids [16]. Social aphids producing morphologically specialized soldiers on secondary hosts are found in the genus *Colophina* of Eriosomatinae and Hormaphidinae [12]. However, *P. bambucicola* produce second instar soldiers inside galls on primary hosts and first instar soldiers on secondary host bamboos, as mentioned above [14]. Although some studies have been carried out on the life history and defensive behavior of *P. bambucicola* [10,11,13,17,18], most of them have been based on laboratory observations and experiments. To our knowledge, there is no research on the functional morphology of *P. bambucicola* based on natural populations. We think this species is a good model to investigate the functional and morphological linkage between defensive behaviors of aphid soldiers and their specialized body parts. For instance, the hind leg waving of *P. bambucicola* soldiers can be considered deimatic behavior, but until now, it has not been tested whether hind leg waving is functionally supported by longer hind legs. Therefore, this study aims to understand the functional morphological basis of defensive behaviors of *P. bambucicola* by testing whether soldiers exhibit morphological specialization for specific behaviors. Field observations and collections were undertaken to obtain behavioral data and specimens, and a comprehensive morphometric analysis was carried out to reveal patterns of representative morphological characters.

## 2. Materials and Methods

### 2.1. Field Sampling and Observation

**Figure 1:**
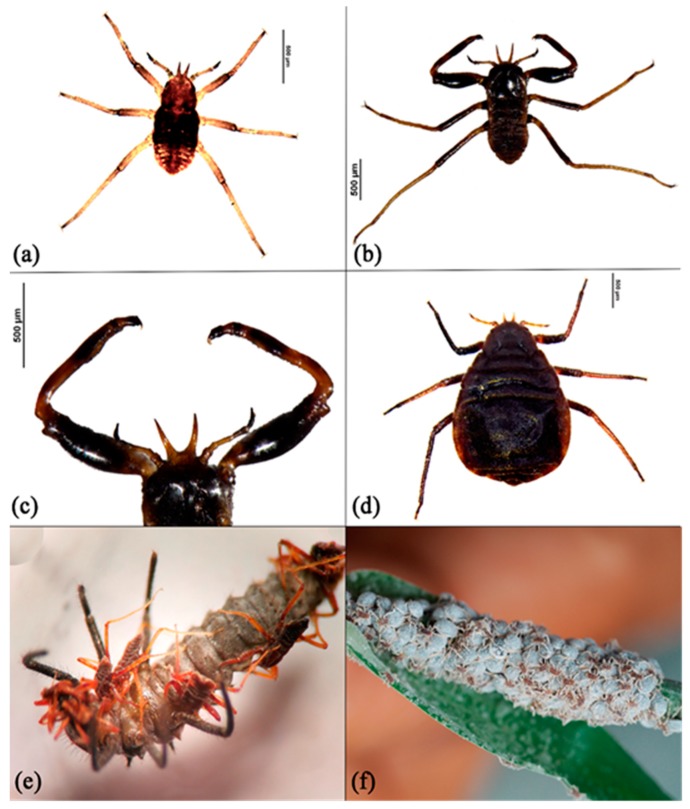
Dorsal view of specimens of *Pseudoregma bambucicola*: (**a**) first instar nymph, (**b**) soldier aphid, (**c**) front legs of soldier aphid, and (**d**) adult. (**e**) Live photo of soldiers attacking *Synonycha grandis* larvae. (**f**) Newly established colony on bamboo shoot.

Field sampling and observations were performed for 62 colonies of *P. bambucicola* on *Bambusa* host plants at different locations in Fujian Province in Southern China, from 2015 to 2017. Usually, a large group of individuals with consecutive distribution on the bamboo stem was considered as one colony (Figure 1f). Aphid samples were collected with the help of a camel-hair brush in the field, and were preserved in 95% alcohol at −20 °C in the lab. Because soldier production fluctuates in different seasons, not all colonies had soldier aphids. In the field, photos of aphid colonies were also taken and videos of behavior were recorded.

### 2.2. Morphometry

For the measurement of individuals of different morphs, we selected first instar nymphs, soldiers, and adults of *P. bambucicola*. Basically, three individuals of each morph were randomly selected from each colony, and the dimensions of head horn (H), body length (BL), body width (BW), front leg (FL), middle leg (ML), and hind leg (HL) were measured (Figure 1a–d). Because the relative proportion of different morphs was different for the 62 colonies, specific morphs (especially the soldier) might be absent from some colonies. In total, a finalized dataset was based on the measurements of 183 first instar, 101 soldier, and 186 adult aphids. Entire lengths of body parts of every individual of each morph were measured with a calibrated eyepiece on a stereomicroscope.

### 2.3. Statistical Analysis

To test the morphological differences and correlations between different morphs, we performed principal component analysis (PCA) on the morphometric data, consisting of body width, body length, front leg length, middle leg length, hind leg length, and frontal horn length, using R studio. PCA abstracts the key mathematical principal components (PCs) from a vast data matrix, by transforming them into two smaller matrices that are easy to understand, examine, and interpret. The first PC indicates the largest portion of the variance in the morphological data, and the second PC explains the second-largest portion of the variance. The results of PCA are generally displayed as score plots (indicating the significance of each sample in a principal component) and biplots (displaying both samples and variables for two principal components). A biplot uses points to signify the scores of the measurements on the principal components, and employs vectors to represent the coefficients of the variables on the principal components. Here, correlated variables and samples were located in the same areas on a biplot. Bivariate scatterplots were constructed to analyze trait relationships and the computation of descriptive statistics. All aspects of statistical analysis were undertaken using the IBM Statistical Package for the Social Sciences (SPSS) version 22.0 (Chicago, IL, USA).

## 3. Results

*P. bambucicola* clustered at different sections of bamboo plants, especially on newly growing shoots (Figure 1f). As their colonies grew, many natural enemies attacked them. Our field observations clearly showed that the leg-waving deimatic behavior of *P. bambucicola* (see supplementary Video S1) was used to prevent predator attacks. When predators attacked, soldier aphids showed aggressive behavior and attempted to insert their frontal horns into them (see supplementary Video S2). It was also observed that predators with a larger body size were often attacked by a larger group of soldiers (Figure 1e). The soldiers usually attacked the legs of large predators as if they were trying to topple the predators from the bamboo (see supplementary Video S2). The morphological variations between the body parts of soldiers, first instar nymphs, and adults of *P. bambucicola* can be seen in Figure 1a–d.

**Figure 2:**
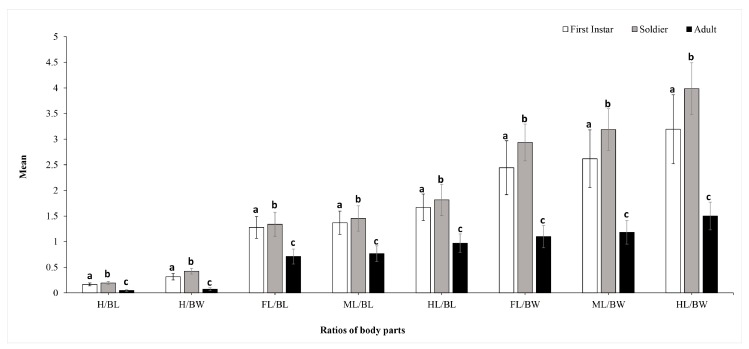
The comparison of body part ratios of different morphs of *P. bambucicola*. Letters on bars mean significant differences (*p* < 0.001).

We identified, separated, and measured 183 first instars, 101 soldiers, and 186 adults from 62 colonies of *P. bambucicola*. The homogeneity of variance test (multivariate General Linear Model GLM analysis) showed that soldiers were morphologically different from first instar nymphs and adult aphids. Morphological variations presented significantly among first instars, soldiers, and adults of *P. bambucicola* (F_72,343,766_ = 30.852; *p* < 0.001). Figure 2 shows that soldiers were morphologically distinct from first instars and adult aphids. Larger ratios of body parts were observed for soldiers compared with first instar normal individuals.

**Figure 3:**
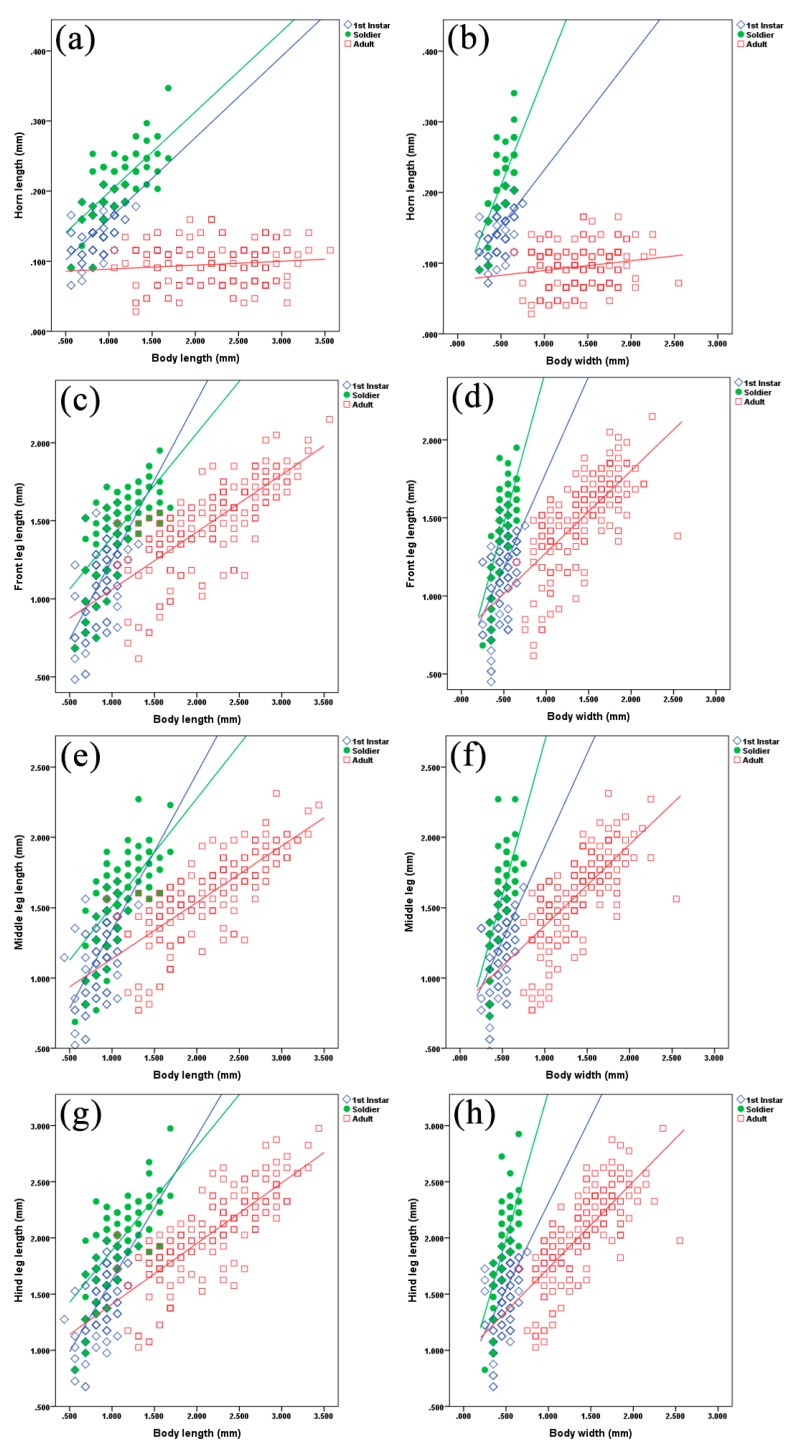
Bivariate scatterplots based on the measurements of different body parts of *P. bambucicola* first instars, soldiers, and adults. (**a**) Horn length vs. body length; (**b**) horn length vs. body width; (**c**) front leg length vs. body length; (**d**) front leg length vs. body width; (**e**) middle leg length vs. body length; (**f**) middle leg length vs body width; (**g**) hind leg length vs. body length; (**h**) hind leg length vs. body width. The open blue diamonds (◊) indicate first instar nymphs, the green circles (●) indicate soldiers, and open red squares (□) indicate adults.

Multiple linear regression models revealed the evolutionary patterns of body part development for first instar nymphs, soldiers, and adult normal aphids, as well as the relationships between them. The scatterplot in Figure 3a shows a good linear relationship between the horn length and body length of first instar nymphs and soldiers, but adults showed a weak relationship. Figure 3b shows a good linear relationship between the horn length and body width of first instar nymphs, while soldiers showed a slightly less linear relationship than first instar nymphs. Figure 3c–g illustrates a positive correlation among body parts, which means that an increase in one variable causes a positive increase in other variables. These scatterplots showed that adults are developmentally different from first instars and soldiers. Some overlapping values for first instars and soldiers indicated that they are morphologically alike in body length and width, but different in terms of frontal horns and front, middle, and hind legs, with respect to body length (Figure 1b,d,f,h). This also demonstrates that soldiers have more well-developed sclerotized horns and front and hind legs than first instars, which demonstrates that horn length increases more than body width in soldiers. Moreover, no correlation was observed between the horns and body length of adults (Figure 3a). These morphological variations suggest that the morphologically well-equipped soldiers perform defensive duties in the colony.

**Figure 4:**
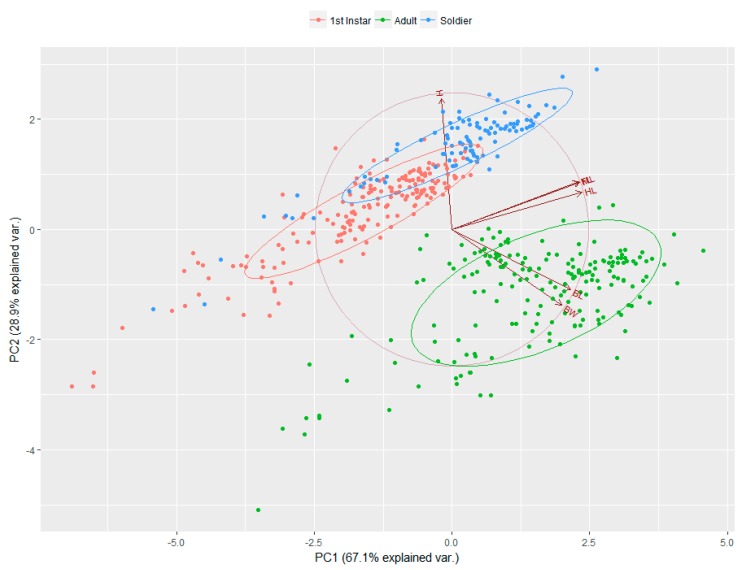
Principal component analysis (PCA) biplot based on the measurements of key morphological characters of *P. bambucicola* morphs. The three different colors (orange, green, and blue) indicate the first instars, adults, and soldiers, respectively. The *x*- and *y*-axes represent the principal component (PC) factor loadings, and the secondary *x*- and *y*-axes represent the PC factor scores. Principal component 1 (PC1, 67.1%) and principal component 2 (PC2, 28.9%) collectively explain 96% of the variation in the dataset.

The PCA biplot elucidated almost 96% variance by the first two components. The first principal component accounted for 67.1% and the second principal component for 28.9% of the variance (Figure 4). Clearly, first instar nymphs and soldiers overlap to make one group, which is separated from adults by the second component. The adults are characterized by high values associated with the BL and BW variables, and low values associated with the FL, ML, and HL variables. The H variable partially separates first instar individuals and soldiers. The variables FL, ML, and HL had a slight angle with all three groups, indicating they were highly correlated. There was a wide angle between the H; FL, ML, HL; and BL, BW variables, which indicates that these groups were independent (regarding linear correlation coefficient).

## 4. Discussion

Our results revealed the morphological differences between first instar nymphs, soldiers, and adults of *P. bambucicola*. It is evident that the cost of morphological specializations of soldier aphids, such as longer horns and hind legs (Figure 3), may affect their body width. The high variation observed in the sizes of horns and legs in soldiers and other morphs indicates that use of these morphological characters may be the best way to differentiate soldiers from first instars and adults of *P. bambucicola*. Our results agree with the results of Stern et al. [17], who state that the soldiers of *P. bambucicola* have elongated horns and legs relative to body size in comparison with other morphs. It has been strongly suggested that soldiers defend their colonies from natural enemies by using their specialized body parts [19]. Soldiers of *Pseudoregma* can easily crush the eggs of *Eupeodes confrater*, which are laid directly onto bamboo shoots, by using their head horns [11,20,21]. According to Ohara [21], a single soldier of *P. bambucicola* can lift up a newly hatched larva of *E. confrater*, and may drop the colony together with the larva. They effectively protect the colony from *Synonycha grandis* (Coccinellidae), *E. confrater* (Syrphidae), and other natural enemies by killing or removing them [20].

The soldier cast might be costly for eusocial aphids [22]. Producing soldiers requires higher nutrition and more maintenance than normal aphids, and may also lower the natural growth rate [23,24]. The population density, size, and age of colonies, as well as the nutritional condition of the host plants, may also affect soldier production. Soldier production is too costly for mothers because of the reduction of fertile progeny [13]. Many insect species regulate soldiers according to biological and environmental cues [22,25]. The production of soldiers in *P. bambucicola* was observed to increase in autumn and decrease in summer, and more soldiers were observed in larger populations [10]. Massive colonies attract more natural enemies, which reduces ant attendance rates but triggers a higher soldier production rate [13]. Polymorphism is triggered by various cues, such as crowding, temperature, and day length [26]. Soldier production in response to numerous environmental and biological cues can amplify defense, colony fitness, and soldier maintenance at minimal fitness costs [16,27].

Not only does the eusocial lineage have a cost, but soldiers also have to pay the price for ensuring colony fitness. They input maximum energy into defensive body parts, as opposed to body size. Sterility in soldiers [10,13,17,18] might be involved in triggering physical specialization and behavioral plasticity. This cast is determined in the embryonic developmental stage [28]. Soldier aphids never molt, and remain in the same instar for a prolonged period of time or throughout their lives in eusocial aphids [6,19]. It is believed that the adaptations of morphology, behavior, and discrimination of the soldier cast play a significant role in the benefits of social life, and necessarily, the success of the evolution of eusociality [22,29]. For example, the soldiers of gall-producing social aphids are dynamically involved in gall resorting and cleaning, such as removing shed skins, carcasses, and deserted honeydew, as well as repairing the openings and damage done by predators [12,30,31].

The PCA results showed clear morphological and functional differences between first instars, soldiers, and adult aphids. According to Figure 4, first instars and soldiers are alike, but have slight morphological differences in terms of front legs and front horns; in contrast, the adults are different morphologically and functionally. First instar nymphs also have relatively long horns, which decrease in successive periods [32]. Early instar nymphs have long legs, so they can actively walk on bamboo shoots when disturbed. The extended front and hind legs of first instars and soldier aphids are used to scare their natural enemies by waving [13]. According to our results, the growth patterns of soldiers are different than other morphs, such as the increase in horn and front leg lengths being positively correlated with their body length rather than body width (Figure 3a–d). It can be clearly seen that the soldiers have robust horns and front legs, along with body width. Morphologically specialized soldiers evolved differently in some genera of Hormaphidinae and Eriosomatinae. For example, first instar soldiers of *Colophina* (Eriosomatini: Eriosomatinae) on secondary hosts have enlarged fore and mid-legs, as well as reduced frontal horns [33]. First instar soldiers of *Pemphigus spyrothecae* (Pemphigini: Eriosomatinae) in the galls on primary hosts have enlarged hind legs [34]. First instar soldiers of *Ceratovacuna* and *Pseudoregma* (Cerataphidini: Hormaphidinae) on secondary hosts have enlarged forelegs and long frontal horns [32].

Deimatic display is a counter-defense or a preventative defense triggered by stimuli [2]. Many insects, such as mountain katydids, praying mantises, phasmids, butterflies, and moths, display deimatic behavior. These insects are cryptic in coloration, and expose themselves in response to stimuli [35]. Some insects make severe sounds and inaudible vibrations, or feign death to avoid predation. A recent study reported that feigning death has been observed in a number of aphid species, especially in the subfamily Lachninae [36]. Some insects mimic an unfavorable and dangerous organism, such as a scorpion (e.g., phasmids) [3,37,38] or slightly eusocial soldier aphids (Figure 1b). There has been only one example of deimatic coloration found in Hemipteran insects, which is the spotted lanternfly (*Lycorma delicatula*), which displays defensive behavior against natural enemies [5]. However, we have not encountered any example of deimatic behavior in aphids. Although deimatic coloration is not present in aphids, leg waving can be considered as deimatic display behavior. As a primary defense, soldier aphids use their sclerotized horns and front legs to attack enemies, and as a secondary defense, they wave their hind legs to scare enemies. Dixon [26] observed the leg-kicking behavior by *Aphis fabae* as a defensive behavior against *Adalia decempunctata*. Leg-waving behavior is mainly found in social aphids of the subfamily Hormaphidinae [10,13,17]. Soldiers and other nymphs of *P. bambucicola* wave their fore or hind legs in reaction to mechanical stimuli [13]. The leg-waving behavior of *Pseudoregma* soldiers has also been observed by other researchers. For example, Stern et al. [17] report the leg waving of *Pseudoregma* sp. on bamboo when predatory wasps fly or walk over the colony. Aoki and Kurosu [14] observed *P. bambucicola* soldiers reacting to the proximity of female *E. confrater* by lifting and waving their hind legs. The deimatic leg-waving behavior of *P. bambucicola* might be a kind of bluff that is successful against their natural enemies (e.g., predators, parasitoids, and birds). Considering that genetic divergence may exist among different populations of *P. bambucicola*, for future studies it will be interesting to investigate in detail whether defense allocation can be affected by genetic divergence. and to show population or colony variations across a large geographic region.

## 5. Conclusions

Our study found that social aphids (*P. bambucicola*) show deimatic behavior towards their natural enemies. They produce morphologically distinct, sterile first instar soldiers to protect their colonies. Morphometric analysis revealed variations in the defensive body parts of soldiers and other morphs, namely sclerotized front legs and robust head horns. Our results clearly showed that first instar soldiers and normal first instar nymphs are somewhat alike, but have a few morphological differences in their front legs and front horns; in contrast, adults are completely different morphologically and functionally. Field observations and a comprehensive morphometric analysis helped elucidate the important functional morphology of deimatic hind leg waving, sclerotized front legs, and head horns. Body size variations also demonstrate that the aphids can perform various functions, such as elongated leg shaking or waving as deimatic behavior and direct attacks on different natural enemies by soldiers using sclerotized front legs and horns. The size variation of different body parts among different morphs also indicates a cost–benefit trade-off in the evolution of social aphid species. This study provides a different and new idea for further studies on aphid deimatic behavior. Moreover, comprehensive studies on the functional morphology of eusocial aphids are needed to provide a complete understanding of the interactions between different structures and behaviors.

## Supplementary Materials

The following are available online at https://www.mdpi.com/2075-4450/10/6/163/s1. Video S1: Video illustrating the leg-waving deimatic behavior of *Pseudoregma bambucicola* in response to stimuli perceived as a threat to their colony, Video S2: Video illustrating soldiers attacking the larvae of *Synonycha grandis*.
