# Supplementary material for: Functional Morphology and Defensive Behavior in a Social Aphid
Source: Insects. 2019 Jun 9;10(6):163. doi: 10.3390/insects10060163 (PMC6627702; doi:10.3390/insects10060163)
Supplement: Supplementary file 1 [file insects-10-00163-s001.zip › Caption of supplementary videos.doc]

**Caption**

**Supplementary Video S1:** Video illustrating the leg-waving deimatic behavior of *Pseudoregma bambucicola* in response to stimuli perceived as a threat to their colony*.*

**Supplementary Video S2:** Video illustrating soldiers attacking the larvae of *Synonycha grandis.*
